# Achieving high energy absorption capacity in cellular bulk metallic glasses

**DOI:** 10.1038/srep10302

**Published:** 2015-05-14

**Authors:** S. H. Chen, K. C. Chan, F. F. Wu, L. Xia

**Affiliations:** 1 Advanced Manufacturing Technology Research Centre, Department of Industrial and Systems Engineering, The Hong Kong Polytechnic University, Hung Hom, Kowloon, Hong Kong; 2 School of Materials Science and Engineering, Liaoning University of Technology, Jinzhou, 121001, China

## Abstract

Cellular bulk metallic glasses (BMGs) have exhibited excellent energy-absorption performance by inheriting superior strength from the parent BMGs. However, how to achieve high energy absorption capacity in cellular BMGs is vital but mysterious. In this work, using step-by-step observations of the deformation evolution of a series of cellular BMGs, the underlying mechanisms for the remarkable energy absorption capacity have been investigated by studying two influencing key factors: the peak stress and the decay of the peak stress during the plastic-flow plateau stages. An analytical model of the peak stress has been proposed, and the predicted results agree well with the experimental data. The decay of the peak stress has been attributed to the geometry change of the macroscopic cells, the formation of shear bands in the middle of the struts, and the “work-softening” nature of BMGs. The influencing factors such as the effect of the strut thickness and the number of unit cells have also been investigated and discussed. Strategies for achieving higher energy absorption capacity in cellular BMGs have been proposed.

Mechanical energy dissipation is crucial in natural structural materials, such as the bones^1^,^2^,^3^, nacres^4^,^5^, and dermal armors^6^,^7^. To dissipate more mechanical energy, it requires that the material has both a high strength so as to increase the threshold for crack initiation and a high toughness to subsequently shield the propagation of the crack. Unfortunately, these two properties are mutually exclusive in most materials^8^. To synthetically fabricate materials with higher energy dissipating efficiency is challenging. Up to now, there are some research directions to increase the mechanical energy dissipation, for instance, to develop artificial hybrids mimicking the complex hierarchical microstructures of bones and nacres^4^,^9^,^10^, where the ceramic/polymer composites are able to demonstrate both high strength and toughness^9^. To change the elastic properties by tuning the chemistry 11 or to fabricate pre-existing shear bands^12^ can also improve the toughness of some amorphous alloys significantly. Besides, a relatively mature technology has been widely used, i.e., introducing micro pores into metals to dissipate more mechanical energy by forming a steady plastic-flow plateau stage, which is beneficial for lightweight and energy-absorption purposes^13^,^14^,^15^.

In the past decade, bulk metallic glasses (BMGs), having nearly ideal strength that is superior to their crystalline counterparts^16^,^17^,^18^,^19^,^20^, have been used to synthesize BMG foams^21^,^22^,^23^,^24^,^25^,^26^,^27^ and honeycombs^28^,^29^ to achieve better energy-absorption performance. Although BMGs are brittle in their bulk form, fabricated BMG foams/honeycombs have demonstrated large room-temperature plasticity, exhibiting great energy-absorption potential^28^,^29^,^30^,^31^. Inheriting high strength from the parent BMGs, BMG foams and honeycombs have demonstrated higher energy absorption capacity than conventional metal foams^28^,^29^,^32^,^33^. BMGs have become a new class of potentially parent materials for designing and fabricating cellular materials, aiming for high energy-absorption applications. More recently, a new kind of cellular BMGs constructed using macroscopic cellular structures rather than microscopic pores have been designed, demonstrating substantially enhanced energy absorption capacity as compared with the BMG foams and honeycombs^34^. The findings imply that designing cellular BMGs with macroscopic cellular structures could be a possible answer for enhancing the energy-absorption performance of cellular solids. However, all these studies remain a question, i.e., how to achieve better energy-absorption performance in cellular BMGs? In this work, by revealing the mechanisms for the remarkable energy absorption performance of a series of cellular BMGs, strategies for achieving high energy absorption capacity in cellular BMGs have been proposed. The cellular BMGs, consisting of macroscopic cellular structures rather than microscopic pores, investigated in this work are selected for the following reasons (1): they have better energy-absorption performance than the existing BMG foams and honeycombs; and (2) they have periodic macroscopic cellular structures for easier establishing an analytical model of the peak stress.

## Results

### Compressive response

**Figure 1:**
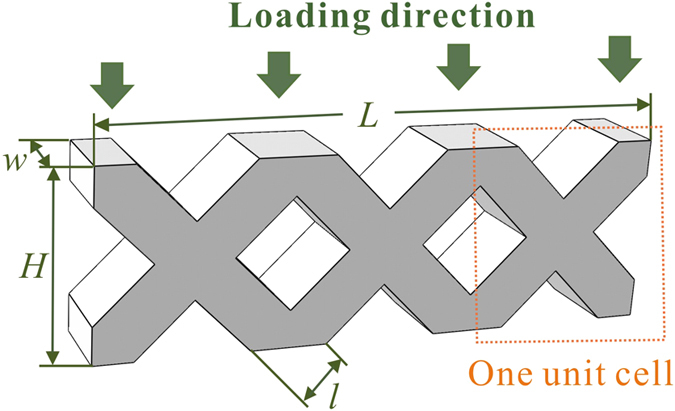
Schematic illustration showing the designed cellular BMGs, where *H* = 3.96 mm, *w* = 2.0 mm, *L* = 11.88 mm, and *l* is the strut thickness^34^.

**Figure 2:**
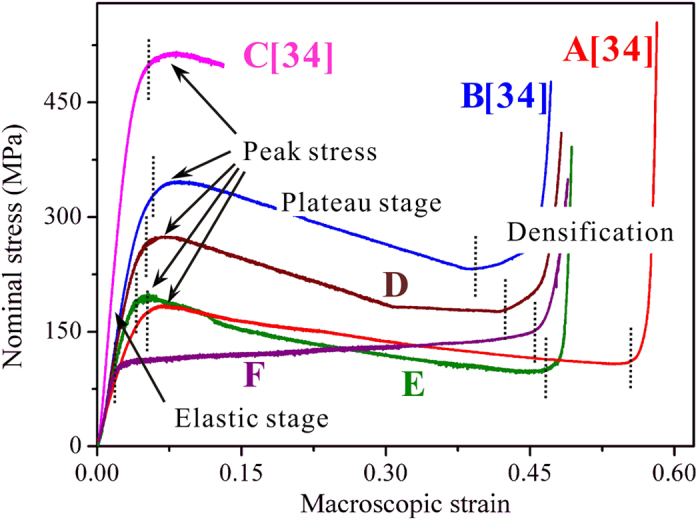
The compressive responses of the cellular materials.

According to the schematic illustration in Fig. 1, a series of cellular BMGs (Table 1) with relative densities of 0.67 (strut thickness *l* = 1.2 mm), 0.59 (*l* = 1 mm) and 0.49 (*l* = 0.8 mm) have been fabricated^34^. The nominal compressive stress-strain curves of the cellular specimens are shown in Fig. 2. It can be seen that, besides specimen C, all the specimens have three stages of deformation: the elastic stage, the plateau stage and the densification stage. The magnitude of the peak stress relates to not only the relative densities (specimens A-C) but also the number of unit cells (specimens B, D and E). During the plateau stages, all cellular BMGs have decreasing peak stresses till the onset of the densification stage. In contrast, the cellular structure made from stainless steel (specimen F) exhibits a different phenomenon: no peak stress was obtained after the elastic stage, and the load increased slightly during the plateau stage.

### Energy-absorption performance

The energy absorption per unit volume (*W*_v_) of the cellular specimens is estimated using the equation below^35^
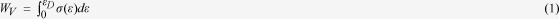
where *σ*(*ε*) is the nominal stress and *ε*_D_ the nominal strain at densification. The calculated values are listed in Table 1. It has been found that the energy absorption capacity of cellular BMGs is significantly affected by the relative densities and the number of unit cells. The increase of the *W*_v_ value, as the relative density increases, is easily understood and common in cellular metals^15^,^34^. However, it is interesting to find that, with same relative density (specimens B, D, and E), the decrease of the number of unit cells results in a decrease in the energy absorption capacity. In order to uncover the energy-absorption mechanisms in cellular BMGs, the deformation evolution in the struts and nodes was investigated.

### Deformation evolution in the cellular BMGs

**Figure 3:**
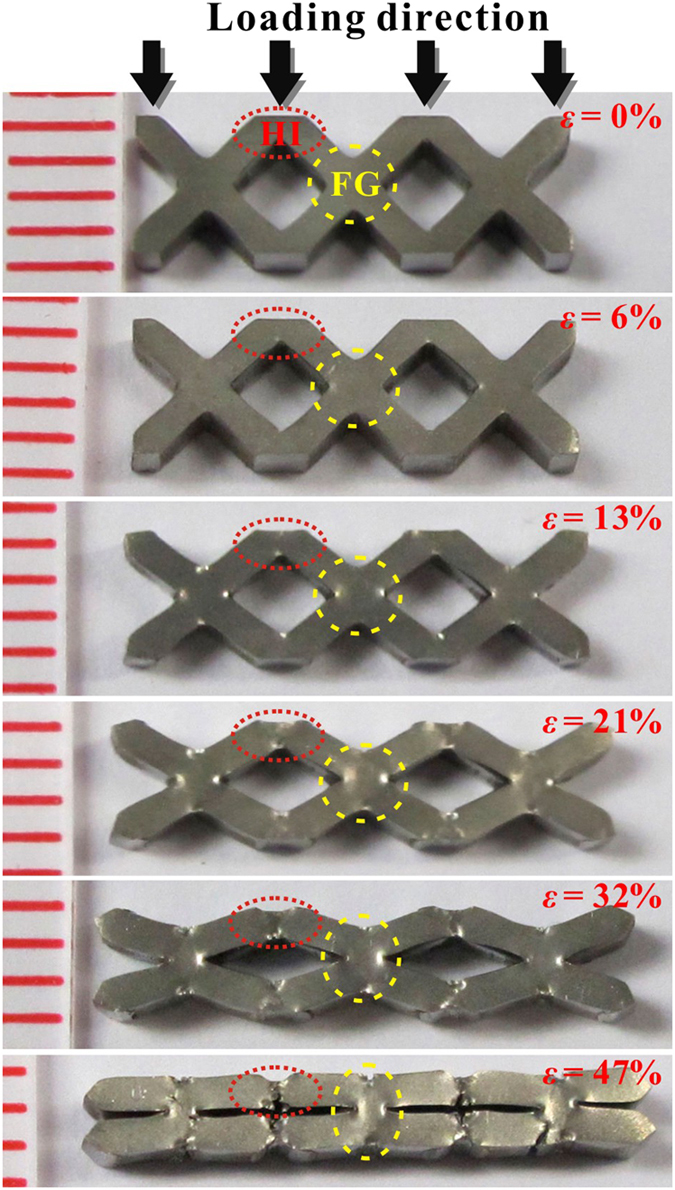
Optical images of a cellular BMG (specimen B) at different macroscopic strains.

**Figure 4:**
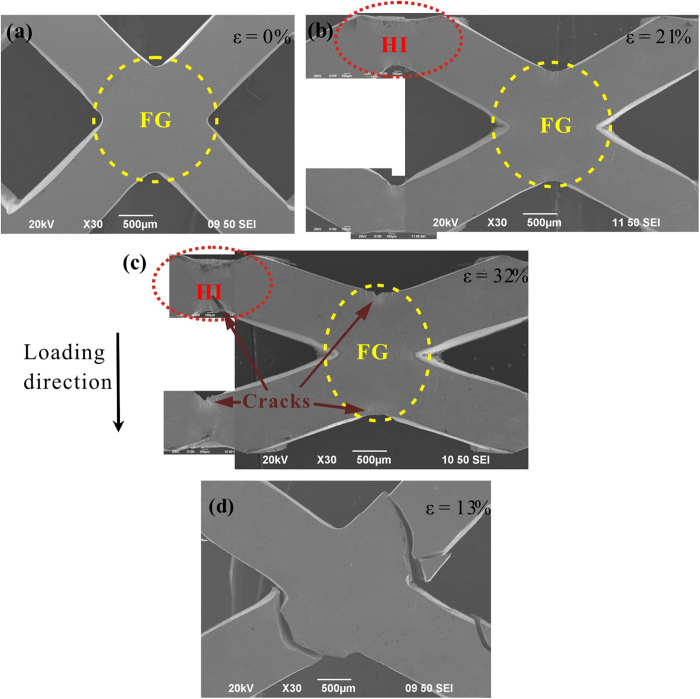
The deformation evolution of the struts in a specimen B at various nominal strains (**a**–**c**). (**d**) An SEM image of a fractured specimen C.

The deformation evolution in a typical specimen (B), from undeformed to densification, is shown in Fig. 3. During the densification process, plastic deformation in the cellular specimen is found to be mainly localized at the nodes (region FG) and half-nodes (region HI). No global failure was observed and the struts were significantly bent after densification. The macroscopic square-shaped cells were compressed to a diamond shape and then were nearly eliminated after densification (*ε* = 47%). Figure 4a–c) shows the magnified SEM images of the deformation evolution in the struts and nodes of specimen B in Fig. 3. It can be seen that the struts tilted at 45° in the undeformed specimen (Fig. 4a)) were significantly bent during the loading process (Fig. 4b,c). Cracks were initiated at the stress concentrated corners in regions HI and FG during the densification process, as can be seen in Fig. 4c). Intrinsically, the high toughness of the parent ZrCuAlNiTi BMG, with a plastic zone radius of about 0.6 mm^34^, enables the crack propagation to be shielded to achieve a wider plastic-flow plateau stage^36^. In specimen C (*l* = 1.2 mm), the cracks nucleated at region FG caused catastrophic failure (Fig. 4d). This might have resulted from the size effect of the BMGs in that the specimen with a thickness larger than 1 mm has a relatively poor bending plasticity^34^,^37^. Macroscopically, the cellular BMGs exhibited large compressive plasticity, via the large plastic bending of the struts. Since the plastic deformation in cellular BMGs is mainly localized in regions **HI** and **FG**, the large plastic bending of the struts is believed to result from the large plastic deformation at the nodes. It worth mentioning that, although a few scattered shear bands have also been observed in some struts at nominal strains larger than 6%, these struts maintain their original straight shapes during the densification process, suggesting that these shear bands may not affect the plastic bending of the struts. Our further investigations have demonstrated that these shear bands influence the peak stress evolution during the plastic-flow plateau stages, and are presented in the discussion section.

**Figure 5:**
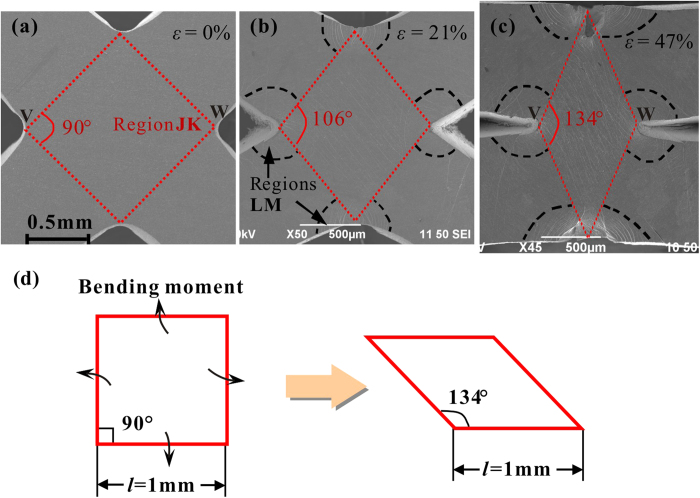
(**a**–**c**) are the SEM images showing the plastic deformation of a node in the specimen B at different nominal strains, and (**d**) is a schematic diagram showing the shape changes of the node (region JK).

The plastic deformation at one of the nodes (region FG in Fig. 4) is magnified in Fig. 5. It can be seen that the square-shaped node (region JK) finally changes to a diamond shape with a angle of about 134°. It can be deduced that, under compression, four bending moments act at the node, and result in the changes of the shape, as illustrated in the schematic diagram in Fig. 5d. Considering the geometric changes of region JK (Fig. 5d), the shear strain is estimated as *γ* = 44° (0.77 rad), which is substantially larger than the fracture value of a Zr-based BMG obtained from torsion tests (0.45 rad)^38^. After densification (at *ε* = 47%), the distance between two opposite vertices (indicated as V & W in Fig. 5) of region JK can be calculated as *d*_den_ = 0.78 mm. The distance changes during the deformation process can then be estimated as *d*_change_ = *d*_ori_ − *d*_den_ = 0.63 mm, where *d*_ori_ is the distance before deformation (Fig. 5a). This illustrates that, during the densification process, the node has undergone large plastic flow under the four bending moments. The changes of the node shapes as well as the large shear strain imply that severe plastic deformation occurs in this region. In addition, at the four corners of the node, other regions (LM) with dense shear bands are also observed. The shear-band region characteristics at the nodes validate the assertion that the large plastic bending of the struts, related to the higher energy absorption capacity of cellular BMGs, is due to the severe plastic deformation at the nodes.

## Discussion

### Analytical modelling of the peak stress

For cellular materials, it has been reported that the peak stress (*σ*_peak_) of the cellular structures scales linearly with the yield stress of the parent material (*σ*_y_), depending on the relative density (*ρ*_r_):^15^
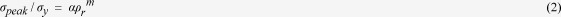
where *α* and *m* are scaling parameters relating to the cell structures, such as *α* = 0.3 and *m* = 1 for the periodic tetrakaidecahedron. In present macroscopic cellular BMGs, it is clear that the peak stress of the cellular BMGs with same relative density (specimens B, D and E) also depends on the number of unit cells (see Fig. 2 and Table 1). Therefore, to predict the peak stress of cellular BMGs, we need to propose a new analytical model, which not only shows the effect of the relative density (strut thickness) but also captures the effect of the number of unit cells.

The differences of the mechanisms, in dissipating the mechanical energy between the cellular BMGs and the metal foams, result from the macroscopic cellular structures. In foam structures with micro cells and pores, the yielding occurs in unit cells and due to the small size and the large number of the unit cells, the peak stress is not sensitive to the change of the macroscopic specimen dimensions, but it is dependant on the relative density^15^. However, in cellular BMGs with macroscopic cellular structures, when peaking of the stress occurs, yielding is found to be only localized at the nodes. For specimen E, after peaking, yielding only occurs in regions JK and LM. For the specimen with more unit cells, shear bands are also distributed in region HI (as can be seen in Fig. 4). Therefore, in the present work, we relate the peak stress of the cellular BMGs to the yield regions, which is significantly affected by the strut thickness and the number of unit cells. Assume that (1) the area of region HI (Figs. 3 and 4) is half the value of region FG (regions JK+4LM in Fig. 5) (2) four regions LM (Fig. 5) form two circular regions with a radius of *l*/4, and (3) when the cellular BMGs reach the peak stresses, the structures maintain their original shapes, i.e., their struts still have a tilt angle of 45°. We propose that the linear relationship (*σ*_peak_ /*σ*_y_) is proportional to the ratio (the yield volume)/(the volume of cellular BMGs). The relationship is expressed as
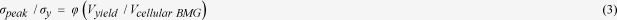
where *φ* is a constant relating to the macroscopic structures. In the present work,
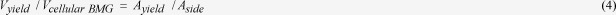
where *A*_yield_ is the total area of the observed yield regions JK and LM on the side surface, and the *A*_side_ is the area of the side surface of the cellular BMGs. For a cellular BMG with *n* unit cells,
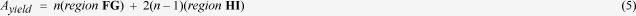


Based on the assumptions (1) and (2), and substituting the geometrical parameters to Equation (5), the area of the yield regions (*A*_yield_) on the side surface of the cellular BMG can then be presented by
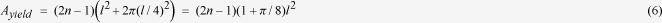


The area of the side surfaces
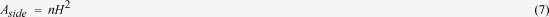
where *H* is the height of the cellular BMGs. Combining the Equations (3, 4, 5, 6, 7), we obtain
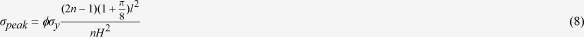


**Figure 6:**
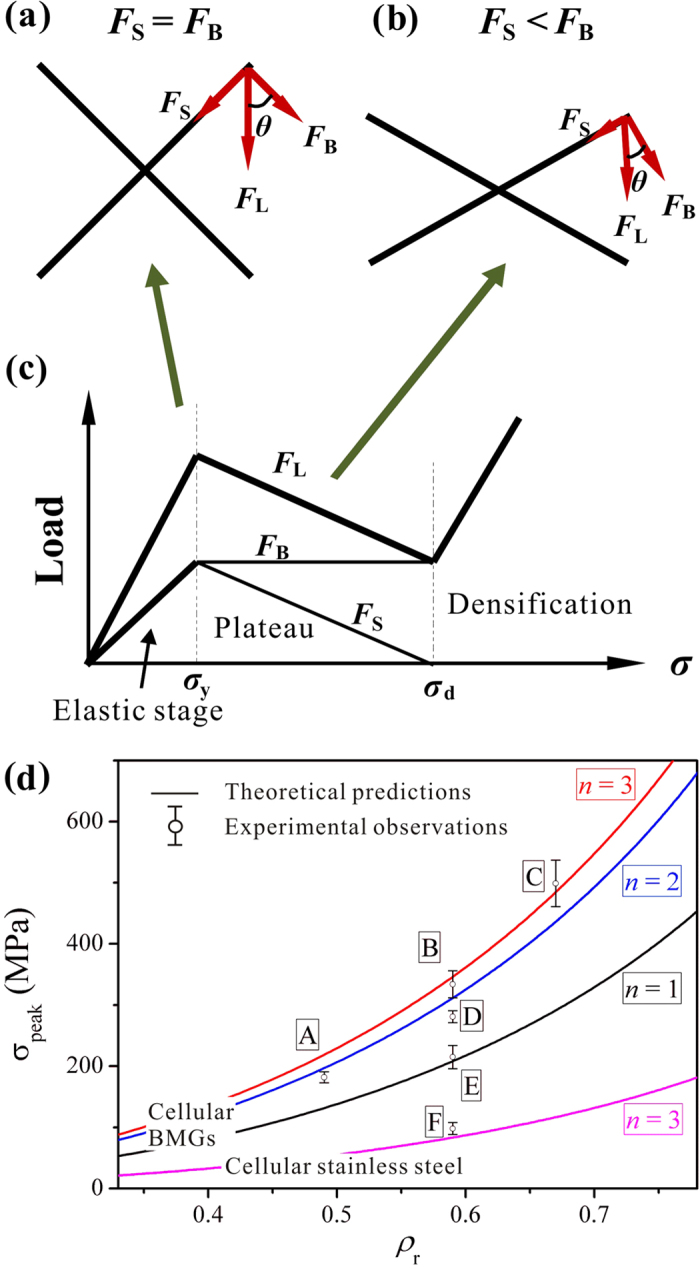
Schematic diagram showing the evolution of the supporting load (*F*_L_) of the cellular structures (**c**) and the corresponding geometry change of one unit cell, where (**a**) is at the peak stress and (**b**) during the densification stage. (**d**) A comparison between the experimental and the theoretical (according to Equation 8) peak stresses of the cellular structures.

Figure 6 shows a schematic diagram of the evolution of the struts in one unit cell. With the assumption that, at the nominal strain where peak stress occurs, the cell maintains the original geometry (Fig. 6a), the stress along the strut (*F*_S_) is related to the compressive load (*F*_L_), i.e., *F*_S_ = sin(*θ*)*F*_L_. Assuming that the yielding of the nodes is caused by *F*_S_ and that *F*_S_/*σ*_y_ = *F*_L_/*σ*_peak_, the constant *φ* is then determined as *φ* = 1/sin(*θ*) = √2, in this work. The calculated results of the peak stresses (*σ*_peak_) of the cellular BMGs are listed in Table 1. The comparison between the predicted results according to Equation (8) and the experimental observations has been plotted in Fig. 6d. It can be seen that the predictions are roughly in agreement with the experimental results. More importantly, such a model captures the effect of the number of unit cells and the strut thickness on the varying peak stress values. Taking *σ*_y_ = 391 MPa from the experimental results of the stainless steel, the “peak stress” of stainless steel specimen F is predicted as 82 MPa (for comparison, the peak stress value *σ*_e_ in Table 1 for specimen F is taken from the strain where the onset of the plastic-plateau stage occurs). The calculated value is within the error range of cellular BMGs, suggesting that such a model is also suitable for cellular structures made from stainless steel. In fact, the deformed specimen F after densification also shows a similar plastic bending phenomenon in the struts, indicating that plastic bending could also be the main deformation mode in this specimen.

### Decay of the peak stress

The other factor, which is also very critical in cellular materials for the energy-absorption applications, is to maintain the peak stress during the plastic-flow plateau stage^15^. Despite the greatly enhanced energy absorption capacity, we have observed a decreasing stress in all the cellular BMGs. To characterize the change of the stress magnitudes during the plastic-flow plateau stages, we define a stress decay factor *f*_d_ as
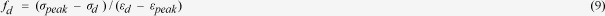
where *σ*_d_ and *ε*_d_ are the nominal stress and strain at the onset of densification. The calculated *f*_d_ values of the specimens are given in Table 1. The decay factor varies with the relative densities and the number of unit cells. Cellular BMGs of *ρ*_r_ = 0.59 have relatively larger values of 2.37-3.38 MPa/1%, decreasing as the number of unit cells decreases, while the specimen with *ρ*_r_ = 0.49 has a decay factor of 1.58 MPa/1%. In contrast, the specimen F, made from stainless steel, has a negative decay factor of -1.05 MPa/1%, indicating that during the plastic-flow plateau stage, the nominal stress increases slightly as compression testing proceeds.

**Figure 7:**
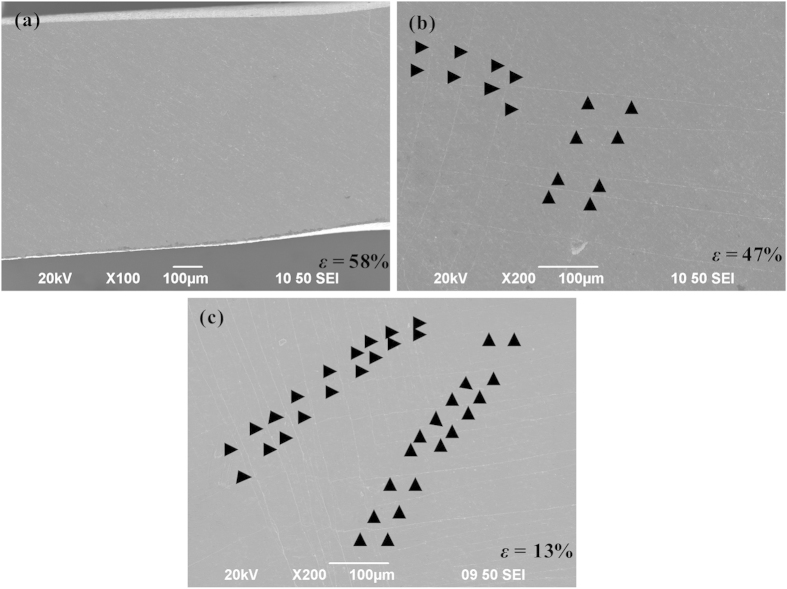
The formation of shear bands in the both ends fixed struts in specimens A (**a**), B (**b**) and C (**c**) respectively, where the triangles indicate the formation of shear bands.

As shown in Fig. 6b, after peaking (i.e., the onset of decay of the peak stresses), the angle *θ* is smaller than 45°. Thus, the magnitude of the component *F*_B_ is larger than *F*_S_, causing the subsequent bending of the struts. As the strut angle *θ* decreases during the plastic-flow plateau stage, if we assume that the load *F*_B_ causing the bending deformation remains constant, it can then be shown that *F*_L_ = *F*_B_/cos(*θ*) also decreases as the loading proceeds. This may explain the partial decay of the peak stress, resulting from the change of the macroscopic structures. On the other hand, examination of the struts in cellular BMGs shows that some shear bands are initiated in the middle of the struts during the plastic-flow plateau stages after peaking (Fig. 7). It is widely recognized that the initiation of the shear bands relates to the corresponding load drops^39^. Although the correlations between the formation of the shear bands in the middle of the struts and the macroscopic applied load appear complicated and are still unknown^40^, it is reasonable to assert that the formation of these shear bands in the middle of the struts can cause the decrease of the applied load. Moreover, the decay of the nominal stresses in cellular structures is also affected by the underlying nature of the parent materials. BMGs are known to have a work-softening effect^41^, while stainless steel has a work-hardening effect. The work-hardening effect can counteract part of the decreasing nominal stresses, even resulting in a slight increase of the loads in specimen F. This also significantly affects the energy-absorption performance of the cellular structures. It was found that although the stainless steel specimen F only has a “peaking stress” of about 106 MPa, the *W*_v_ value still reaches 63 MJ/m^3^, which is comparable to a cellular BMG (specimen E) having a peak stress of about 196 MPa.

### Effect of the strut thickness

The effect of the strut thickness on the energy absorption capacity of cellular BMGs is mainly threefold. Firstly, according to Equation (8), the peak stress of cellular BMGs depends on the strut thickness (*l*). Increase of the strut thickness results in an increase of the peak stress, which will definitely enhance the energy absorption capacity of cellular BMGs. Secondly, the increase of the strut thickness results in the increase of the relative density, which conversely has a larger decay factor (*f*_d_) during the plastic-flow plateau stages, as can be seen in Table 1. This indicates that a larger strut thickness causes a stronger decay of the peak stress. It is noted that the decay factor depends on the macroscopic cellular structure changes, the shear band initiation in the middle of the struts and the “work-softening” nature of BMGs. Since cellular BMGs with various densities have same cellular structure and are made from same as-cast rods, it can be predicted that the formation of the shear bands in the middle of the struts are the likely reasons. This reasoning is verified by the observation of shear bands in the struts. As shown in Fig. 7, in specimen A (*l* = 0.8 mm), no shear band was observed, while in specimens B (*l* = 1 mm) and C (*l* = 1.2 mm), some scattered shear bands were found, and the specimen with the larger thickness has a larger number of shear bands. Thus, increasing the *f*_d_ value in the specimens with larger strut thicknesses can be easily understood, due to the formation of more shear bands (Fig. 7).

Thirdly, the findings show that when the strut thickness increases to about 1.2 mm, catastrophic failure occurs in specimen C at a nominal strain of about 13% (Fig. 4d), resulting from the smaller bending plasticity of the struts^37^. This indicates that there is a critical value of strut thickness for achieving a large plastic-flow plateau stage in cellular BMGs till densification. It should be pointed out that the decrease of the strut thickness can result in a wider plastic-flow plateau, as indicated in Fig. 2. However, due to the greatly decreased peak load, the total energy absorption capacity is still much smaller than the specimens with narrower plateaus. Put simply, besides the catastrophic failure as well as the width of the plastic-flow plateaus, the strut thickness influences not only the magnitude but also the decay of the peak stress of the cellular BMGs. The increase of the strut thickness can enhance the peak stress, but at the same time yield a larger decay of the peak stress at subsequent plateau stages. This contradiction should be minimized and optimized in order to achieve better energy-absorption performance in cellular BMGs.

### Effect of the number of unit cells

As shown in Fig. 2 and Table 1, by comparing the mechanical performance of specimens B, D and E at the same relative density, it was found that the energy absorption capacity decreases as the number of unit cells reduces from three (B) to one (E). Based on the experimental observations, the decreased energy absorption capacity was mainly caused by reducing the peak stresses. We have found that there are no obvious differences in the shear band density at the nodes in these cellular BMGs (B, D and E). If the deformation at the nodes shows no obvious differences, why does the specimen with three unit cells have a larger peak stress? By examining the struts in cellular BMGs at nominal strains of about 6%, where all the specimens have peaked, we found that in specimens D and E, besides the nodes in region FG (Fig. 4), some other half-shaped nodes (region HI in Fig. 4) also have some shear bands. The increase of the nodes with plastic deformation suggests that a larger supporting load may be achieved, causing the increase of the peak stress. This reasoning has further been validated by the good agreement between the predicted values based on Eq. 8 and the experimental observed results, as shown in Table 1.

In addition, the change of the number of unit cells also affects the decay of the peak stress. As shown in Table 1, the decay factor decreases as the number of unit cells decreases. Although the shear band densities in the middle of the struts are affected by the strut thickness, we have observed almost the same shear band densities in the struts with the same thickness. Nevertheless, the study of the formation of the shear bands in the middle of the struts is reminiscent of the fact that the shear band only nucleates in the both ends fixed struts, during the plastic-flow plateau stages. For struts with one end fixed and one end free, no shear band was observed. Specimen B has eight struts with both ends fixed, Specimen D has four, while all the struts in specimen E are fixed at one end with one end free. The ratio of the number of both ends fixed struts to the total number of struts was calculated as 0.67, 0.5, and 0 respectively, for specimens B, D and E. Therefore, despite the same shear band density in the middle of the struts, the increase of the number of unit cells can also increase the decay factor (*f*_d_).

### Achieving better energy-absorption performance

The findings of present cellular BMGs show that the enhancement of the energy absorption capacity is mainly attributed to the increase of the peak stress, which results from not only the high strength of the parent BMGs but also the design of the macroscopic cellular structures. The decay of the peak stress during the plastic-flow plateau stage, conversely, causes the partial neutralization of the increasing effect stemming from the enhanced peak stress. Building on the analytical modelling and the decay factor analysis, to increase the yield volume of cellular structures when peaking occurs and, at the same time, to reduce the decay of the peak stress after the onset of densification are the most efficient routes for achieving better energy-absorption performance. As case in point, for the cellular BMGs in the present work, most parts of the cellular structures such as in the struts are still in an elastic state when peaking occurs. Thus, a larger peak stress may be achieved by tuning the macroscopic cellular structures, including the strut thicknesses and the number of unit cells. Some newly developed techniques, such as 3D printing^42^,^43^, could be used to fabricate cellular BMGs consisting of complex macroscopic cellular structures. On the other hand, BMGs with atomic-scale inhomogeneities^44^ and BMG composites^45^,^46^ exhibiting “work-hardening” behavior might be employed as parent materials in order to reduce the decay of the peak stress.

Before concluding remarks, there are two limitations for the application of the present work which should be pointed out. On one hand, this work only considers one layer of the cellular structures along the perpendicular direction. In practical applications, more layers of cellular structures along the loading direction may be required, and the change of the boundary conditions may affect the deformation behavior of the cellular BMGs. Nevertheless, the present analytical model (Eq. 8) is based on the assumptions that the yield volume of the half-shaped nodes (region HI) is half of the full-shaped nodes (region FG), ignoring the effect of the boundary condition on the deformation of the half-shaped nodes. Thus, the authors believe that the proposed analytical model is still useful on predicting the peak stress of cellular BMGs with more layers. More importantly, the proposed relationship *σ*_peak_ /*σ*_y_ = *φ* (*V*_yield_ /*V*_cellular BMG_) could be expanded to many other cellular BMGs, even though the detailed expression of the analytical model may be changed due to the change of the cellular structures. On the other hand, the application of the macroscopic cellular BMGs may also be hindered by their product size. To use BMGs with larger glass forming ability^47^,^48^ or BMG composites^45^,^46^,^49^,^50^,^51^ which can be easily formed into a larger structure are potential approaches to address the size issue. Furthermore, using a more effective welding process is another approach to synthesize larger BMG structures^52^,^53^,^54^,^55^.

In summary, the mechanisms of the high energy absorption capacity of a series cellular BMGs have been revealed by examining the two determining factors, i.e., the peak stress and the decay of the peak stress at subsequent plastic-flow plateau stages. An analytical model of the peak stress has been proposed, and the theoretically predicted results show high agreement with the experimental results. The decay of the peak stress has been related to the geometric changes of the macroscopic cells, the shear band initiation in the middle of the struts and the “work-softening” nature of BMGs. The findings suggest that, to increase the yield volume of cellular BMGs when peaking occurs by tuning the macroscopic cellular structures including the strut thickness, the number of the unit cells and the tilted angles of the struts might be a promising research direction for achieving higher energy absorption capacity in cellular BMGs.

## Methods

As-cast Zr_57_Cu_20_Al_10_Ni_8_Ti_5_ (at.%) rods of 5 mm diameter were prepared by suction casting the melted mixture of pure Zr, Cu, Al, Ni and Ti into a copper mold. The cellular BMGs were fabricated from the as-cast BMG rods using wire-cut electrical discharge machining (EDM). Compression tests of the cellular structures at a constant loading rate of 0.024 mm/min were performed on a 810 Materials Testing System at room temperature, and the nominal strains were recorded using an extensometer. Three specimens have been tested for each cellular structure. Compressive stainless steel specimens of 3 × 3 × 6 mm^3^ have also been tested at the same conditions for comparison. To investigate the deformation evolution in the struts and nodes, continuous loading of the cellular specimens was terminated at nominal strains of about 6%, 13%, 21%, 32% and 53% respectively till failure (specimen C) or densification (specimens A, B and D-F). After each step of mechanical testing, the side surfaces of the specimens were examined on a Jeol JSM-6490 scanning electron microscope.

## Author Contributions

S.H.C. and K.C.C. designed the research. S.H.C. and F.F.W. performed the experiments. S.H.C., K.C.C. and L.X. analyzed the data, S.H.C. and K.C.C. wrote the manuscript. All authors reviewed the manuscript.

## Additional Information

**How to cite this article**: Chen, S. H. *et al.* Achieving high energy absorption capacity in cellular bulk metallic glasses. *Sci. Rep.* **5**, 10302; doi: 10.1038/srep10302 (2015).

## Figures and Tables

**Table 1:** The fabrication of the cellular structures.

**Label**	**Parent material**	***n***	***ρ***_**r**_	***W***_**v**_**(MJ/m**^**3**^)	***σ***_**y**_**(MPa)**	***σ***_**e**_**(MPa)**	***f***_**d**_**(MPa/1%)**	**Ref.**
A	BMG	3	0.49	56	218	182±9	1.58	34
B	BMG	3	0.59	127	341	334±22	3.38	34
C	BMG	3	0.67	78	491	499±38	—	34
D	BMG	2	0.59	98	307	281±10	2.78	Present work
E	BMG	1	0.59	64	205	215±19	2.37	Present work
F	Stainless steel	3	0.59	63	82	98±10	−1.05	Present work

*n* is the number of the unit cells, *ρ*_r_ is the relative density, *W*_v_ is the energy absorption per unit volume, *σ*_y_ is calculated peak stress, *σ*_e_ is the experimental peak stress, and *f*_d_ the decay factor of the plastic-flow plateaus.
